# Author's reply

**DOI:** 10.4103/0019-5545.49462

**Published:** 2009

**Authors:** Ravi Prakash

**Affiliations:** Department of Psychiatry, Central Institute of Psychaitry, Ranchi, India. E-mail: drravi2121@gmail.com

Sir,

Through this letter, I would first like to thank all the readers of the reputed “*Indian journal of psychiatry*”, who have also read my article – *“The conscious access hypothesis – Explaining the consciousness”.*[1]

Rightly pointed out, I have not elaborated enough the studies of consciousness from the perspective of ancient Indian philosophy. This was because I wanted to present a highly focused “Global Workspace Model of Consciousness”. As I have mentioned in the introduction, the first evidence of any description of consciousness has come from none other than the Indian sacred texts of Vedas, which have vividly described the consciousness as a field phenomena. The words “Aatma” and “Chetna” had definitely the same meanings as the word consciousness that we have today.

Now I turn to a more important question. Why have these concepts not received adequate attention in the modern scientific literature of consciousness? Is it because these are all philosophical or completely abstract? I think not. Rather this represents the absence of the effort to develop a scientific stance towards these states, which is further limited when we see its Indian contributions, which I have mentioned earlier.[2] However, the past decade has evidenced the orientation of scientists across the world for understanding these concepts.

My inquisitiveness lead me to many such Yogic cults with the intention of unraveling their experiences and understandings of consciousness. I found the concepts of two of the Yogic cults specifically interesting in this respect, which are “Kriya Yoga” founded by “Swami Yoganada” and “Vihangam Yoga” founded by Swami “Sadafaldeoji Maharaj”. The following is just a brief overview of what I collected from the vast literature presented in these and other Indian texts.

**Consciousness is a non-material entity:** To start with, the followers of meditation clearly state that the entity of consciousness is nonmaterial in the most subtle (Sookshma) sense.

**Consciousness and attention are different but correlated:** Surprisingly, a matter of recent discussion[4] was clarified long back when Consciousness (Chetna) was differentiated from Attention (Dhyana) since the time these words were coined.[3] It is still conceptualized such in Indian Yogic practices.[4]

**Yogic meditation is a way of understanding consciousness:** The practitioners of mediation speak of several instances when they passed through such states where they could perceive consciousness in a complete form. The firm statements of Vihangam Yogis defining Yoga as the “Science of consciousness” seems to be the result of personal experiences of passing through such states.

**There are several states of consciousness:** Against the current understanding of only normal and altered states of consciousness, Vihangama Yoga followers believe that there are 6 states of consciousness- Sthul awastha, Sukshma awastha, Karana awastha, Mahakarana awastha, Kaivalya awastha and Hamsa awastha.[5] These are the purer states of awareness wherein the consciousness extends beyond body and other limitations that we know of at physical planes. I have appealed else where to explore these states for having objective knowledge rather than discarding such states.[6] Recently we conducted both qualitative study and 192-channel EEG study on the Vihangam Yoga meditation states, from which we found that the meditation is a unique state of peaceful alertness as supported by both subjective statements and objective observation

Through this letter, I am appealing all the inquisitive minds who are in search of consciousness to orient themselves towards these and similar yogic practices. I have received feedback comments from various researchers from different fields of study. If all of them make a collaborative approach towards this topic, the study of consciousness which is an integral part of the Indian heritage, can receive a multidimensional exploration and probably will lead us to the hidden and finer aspects of consciousness.
